# Numerical Modelling of the Mechanical Behaviour of Carbon and Non-Carbon Nanotubes and Their Complex Structures

**DOI:** 10.3390/ma15217515

**Published:** 2022-10-26

**Authors:** Nataliya A. Sakharova

**Affiliations:** 1 Centre for Mechanical Engineering, Materials and Processes (CEMMPRE), Department of Mechanical Engineering, University of Coimbra, Rua Luís Reis Santos, Pinhal de Marrocos, 3030-788 Coimbra, Portugal; nataliya.sakharova@dem.uc.pt; Tel.: +351-239-790-700; 2 ARISE—Advanced Production and Intelligent Systems Associated Laboratory, 4200-465 Porto, Portugal

Systematic research efforts have been focused on the development of low-dimensional structures, such as nanotubes (NTs), because of the potential of their use in nanodevices and in applications in nanoelectronics and biomedicine. After intense investigation on carbon nanotubes (CNTs), nanotubes made up of elements other than carbon, called non-carbon nanotubes (N-CNTs), have also attracted research attention, as they can be more suitable for electronic and optical engineering applications than their carbon counterparts. Success in assembling CNTs and N-CNTs together has opened the perspective of integrating these innovative complex structures into advanced NT-based systems. As experimental studies on the mechanical behaviour of CNTs and N-CNTs are limited by experimental difficulties in characterizing nanomaterials at the atomic scale, theoretical, analytical and numerical studies to predict the mechanical properties of NTs are of great importance.

This Special Issue, comprised of a total of five research articles, is dedicated to recent developments in the modelling and numerical simulation of the mechanical properties of graphene sheets [1]; CNTs and their complex structures [2,3], such as heterojunctions [4]; and, to conclude, boron nitride nanotubes (BNNTs) [5], on which, among other N-CNTs, strong research efforts have been produced so far. The first study by Wang et al. [1] deals with the molecular dynamic (MD) simulation of the Atomic Force Microscopy (AFM) nanoindentation test performed on a single-layer wrinkled graphene sheet to extract its mechanical properties. The deflection of the graphene sheet (GS) under AFM nanoindentation was used to study the effect of wrinkles on the GS’s elastic properties, specifically its Young’s modulus. It was found that surface wrinkling leads to a decrease in the GS’s Young´s modulus. Their results contributed to a better understanding of how to take into account the GSs’ surface morphology to achieve an accurate characterization of the elastic properties of graphene. This is a useful development to study the mechanical response of CNTs, as it can be seen as a rolled-up graphene sheet.

The next two research articles [2,3] of the Special Issue are devoted to multi-walled carbon nanotubes (MWCNTs), which are CNT structures composed of 3 to 50 concentric, single-walled carbon nanotubes (SWCNTs). Sakharova et al. [2] investigated the elastic properties of non-chiral (armchair and zigzag) MWCNTs with up to 20 constituent SWCNTs under tensile, bending, and torsional loading conditions using a simplified finite element (FE) model and without taking into account the van der Waals interactions between layers. The tensile, bending, and torsional rigidities were evaluated. The relationships established between each of the three rigidities and the outer NT diameter constituted the robust methods for assessing the Young´s and shear moduli of the non-chiral MWCNTs. This can be particularly valuable when modelling the mechanical behaviour of MWCNT-reinforced composites and MWCNT-based complex structures is required. In this context, the main goal of the work by Carneiro and Simões [3], who studied the influence of the MWCNTs on the mechanical properties of metal matrix nanocomposites, was to correlate the results, assessed by numerical simulation, with those obtained experimentally and to validate the available methodologies for predicting the strengthening of CNT-reinforced composites. For this purpose, a detailed characterization of the MWCNTs was performed in order to evaluate the inner and outer NTs’ diameters, lengths, number of layers, constituents of MWCNTs, and interlayer distances. Considerable improvement in the mechanical properties, i.e., increases in the hardness values of CNT-based metal nanocomposites, was achieved when straight MWCNTs with large outer diameters were used for reinforcement. From the results, it was possible to validate the existing methodologies, which used MD simulations, developed to predict the mechanical properties of MWCNT-reinforced composites. Moreover, the extensive experimental characterization of the structure and morphology of multi-walled CNTs is mainly useful to feed numerical models of MWCNTs, as described in the work of Sakharova et al. [2].

The research article of Pereira et al. [4] is dedicated to correctly describing the deformation behaviour of SWCNT heterojunctions (HJs), whose structure can be understood as that of two SWCNTs connected by an intermediate region. Therefore, a systematic characterisation of the mechanical properties of armchair–armchair and zigzag–zigzag SWCNT HJs was performed. Three-dimensional (3D) FE simulation was used to carry out a parametric study on the tensile, bending, and torsional rigidities of SWCNT HJs. As a result, analytical methods were formulated for easy assessment of the elastic properties of HJs, in a wide range of their geometrical parameters. As the strength and productivity of the nanodevices and nanosystems strongly depend on the mechanical properties of their components, and CNT HJs are potential candidates for such applications, the work by Pereira et al. [4] represents a substantial contribution to the knowledge on the elastic behaviour of CNTs-based structures.

The last article by Sakharova et al. [5] provides a benchmark with regard to the determination of the elastic properties of single-walled boron nitride nanotubes (SWBNNTs) by numerical and analytical methods. In this study, a systematic characterization of the mechanical behaviour of SWBNNTs was performed for a wide range of chiral indices, diameters, and lengths using the 3D FE simulation method. A comprehensive study of the influence of input parameters on FE modelling of computed elastic properties, such as the tensile, bending, and torsional rigidities, and, subsequently, the shear and Young’s moduli and the Poisson´s ratio of SWBNNTs was also performed. As BNNTs have a great potential to replace CNTs in practical applications and taking into account the perspective of hybrid NT-based structures, consisting of BNNTs and CNTs, the results of this article serve as a guide for the correct design of such novel nanostructures.

The works of this Special Issue promote research for the evaluation of the mechanical properties of carbon and non-carbon nanotubes and CNT-based structures by mostly numerical approaches. As a Guest Editor, I believe that the overall quality of the methodologies and achievements presented in this Special Issue contributes to the progression in our understanding of the mechanical behaviour of carbon and non-carbon nanotubes, aiding the future design and optimization of advanced nanotube-based structures.

## Data Availability

Not applicable.
